# Single-Cell Transcriptome Analysis Highlights a Role for Neutrophils and Inflammatory Macrophages in the Pathogenesis of Severe COVID-19

**DOI:** 10.3390/cells9112374

**Published:** 2020-10-29

**Authors:** Hibah Shaath, Radhakrishnan Vishnubalaji, Eyad Elkord, Nehad M. Alajez

**Affiliations:** 1 College of Health & Life Sciences, Hamad Bin Khalifa University (HBKU), Qatar Foundation (QF), PO Box 34110, Doha, Qatar; hshaath@hbku.edu.qa (H.S.); vbradhakrishnan@hbku.edu.qa (R.V.); eelkord@hbku.edu.qa (E.E.); 2 Cancer Research Center, Qatar Biomedical Research Institute (QBRI), Hamad Bin Khalifa University (HBKU), Qatar Foundation (QF), PO Box 34110, Doha, Qatar

**Keywords:** SARS-Cov-2, COVID-19, neutrophils, inflammatory macrophages, bronchoalveolar lavage (BAL), ICGS2, UMAP

## Abstract

Cumulative data link cytokine storms with coronavirus disease 2019 (COVID-19) severity. The precise identification of immune cell subsets in bronchoalveolar lavage (BAL) and their correlation with COVID-19 disease severity are currently being unraveled. Herein, we employed iterative clustering and guide-gene selection 2 (ICGS2) as well as uniform manifold approximation and projection (UMAP) dimensionality reduction computational algorithms to decipher the complex immune and cellular composition of BAL, using publicly available datasets from a total of 68,873 single cells derived from two healthy subjects, three patients with mild COVID-19, and five patients with severe COVID-19. Our analysis revealed the presence of neutrophils and macrophage cluster-1 as a hallmark of severe COVID-19. Among the identified gene signatures, *IFITM2*, *IFITM1*, *H3F3B*, *SAT1*, and *S100A8* gene signatures were highly associated with neutrophils, while *CCL8*, *CCL3*, *CCL2*, *KLF6*, and *SPP1* were associated with macrophage cluster-1 in severe-COVID-19 patients. Interestingly, although macrophages were also present in healthy subjects and patients with mild COVID-19, they had different gene signatures, indicative of interstitial and cluster-0 macrophage (i.e., *FABP4*, *APOC1*, *APOE*, *C1QB*, and *NURP1*). Additionally, *MALAT1*, *NEAT1*, and *SNGH25* were downregulated in patients with mild and severe COVID-19. Interferon signaling, FCγ receptor-mediated phagocytosis, IL17, and Tec kinase canonical pathways were enriched in patients with severe COVID-19, while PD-1 and PDL-1 pathways were suppressed. A number of upstream regulators (IFNG, PRL, TLR7, PRL, TGM2, TLR9, IL1B, TNF, NFkB, IL1A, STAT3, CCL5, and others) were also enriched in BAL cells from severe COVID-19-affected patients compared to those from patients with mild COVID-19. Further analyses revealed genes associated with the inflammatory response and chemotaxis of myeloid cells, phagocytes, and granulocytes, among the top activated functional categories in BAL from severe COVID-19-affected patients. Transcriptome data from another cohort of COVID-19-derived peripheral blood mononuclear cells (PBMCs) revealed the presence of several genes common to those found in BAL from patients with severe and mild COVID-19 (*IFI27*, *IFITM3*, *IFI6*, *IFIT3*, *MX1*, *IFIT1*, *OASL*, *IFI30*, *OAS1*) or to those seen only in BAL from severe-COVID-19 patients (*S100A8*, *IFI44*, *IFI44L*, *CXCL8*, *CCR1*, *PLSCR1*, *EPSTI1*, *FPR1*, *OAS2*, *OAS3*, *IL1RN*, *TYMP*, *BCL2A1*). Taken together, our data reveal the presence of neutrophils and macrophage cluster-1 as the main immune cell subsets associated with severe COVID-19 and identify their inflammatory and chemotactic gene signatures, also partially reflected systemically in the circulation, for possible diagnostic and therapeutic interventions.

## 1. Introduction

As part of the continuing efforts to further understand the mechanisms underlying viral infection and disease severity, unraveling the role of the immune system is imperative to develop possible therapeutic interventions. The spread of severe acute respiratory syndrome coronavirus 2 (SARS-CoV-2) causing coronavirus disease 2019 (COVID-19) continues to devastate many communities and economies, placing healthcare systems under mass pressures. In cases with severe or fatal outcomes, a hyper-production of cytokines is often observed as a result of an overreaction of the immune system to the infection, causing an imbalance known as cytokine storm [1]. The consequences of a cytokine storm can range from alveolar injury to multi-organ failure, sepsis, or even death. Due to the resulting devastating effects on the host, many studies are now focusing on how cytokine storms can be minimized, as increasing evidence has shown cytokine storms to be in direct association with more severe cases of COVID-19 [2].

Based on previous studies conducted after the emergence of prior coronaviruses, including SARS-CoV-1 and the Middle East respiratory syndrome coronavirus (MERS-CoV), the entry receptor for SARS-CoV-2 was identified as human angiotensin converting enzyme 2 (hACE2) [3]. Viral entry was found to be facilitated through distinct hotspots on hACE2, forming energetically stable tunnel structures for viral binding on hACE2-positive host cells [4]. hACE2 expression on the apical surface of polarized human airway epithelia positively correlates with coronavirus infection, whereas undifferentiated cells expressing little ACE2 had lower rates of infection [5]. Other tissue groups with ACE2 expression, and therefore susceptible to coronavirus infection, include lung, heart, kidney, and gastrointestinal tract [6,7]. Korber et al. reported a SARS-CoV-2 variant harboring the D614G spike protein amino acid change as the most prevalent form in the COVID-19 global pandemic. Interestingly, the authors reported the G614 variant to be associated with higher viral loads in patients but not with increased disease severity [8].

Recognition of a foreign infection triggers innate immune responses leading to mass recruitment of immune cells to the sites of infection. Studies have shown that higher plasma levels of Interleukin 2 (IL2), Interleukin 7 (IL7), Interleukin 10 (IL10), Granulocyte colony-stimulating factor (GCSF), Interferon gamma-induced protein 10 (IP10), monocyte chemoattractant protein 1 (MCP1), Macrophage Inflammatory Proteins 1A (MIP1A), and Tumor Necrosis Factor Alpha (TNFα) were found in intensive care unit (ICU) patients compared to non-ICU patients [9,10], supporting the evidence which attributes severe cases of COVID-19 to an intense immune response. Emerging data have increased our understanding of hallmarks of severe cases of COVID-19. Shi et al. showed a decrease in the number of circulating CD4^+^ cells, CD8^+^ cells, B cells, and natural killer (NK) cells, as well as a decrease of monocytes, eosinophils, and basophils [11]. In addition to this, both human monocytes and macrophages express ACE2 and can be directly infected with SARS-CoV-2, increasing the transcription of pro-inflammatory genes associated with increased COVID-19 severity [1].

Interestingly, Pujhari et al. suggested that more than 70% of COVID-19 deaths are attributed to clotting-associated complications, emphasizing the role of cytokine storms, activation of neutrophils, and release of neutrophil extracellular traps as possible mechanisms contributing to blood clotting disorders [12]. Neutrophilia-induced lung injury in severe COVID-19 patients was also reported by Wang et al., who observed lesions after the second week of disease onset, coinciding with neutrophilia progression [13].

A deep analysis of our complex immune system is important to identify key players involved in COVID-19 severity. Single-cell analysis is therefore a valuable approach that has been utilized to elucidate phenotypic and expressional differences between cell subsets involved in the peripheral immune response in relation to COVID-19 infection [2,14]. In our current study, publicly available single-cell data from bronchoalveolar lavage (BAL) comprising a total of 68,873 single cells derived from two healthy subjects, three patients with mild COVID-19, and five patients with severe COVID-19 were subjected to iterative clustering and guide-gene selection 2 (ICGS2) and AltAnalyze algorithms, identifying gene signatures associated with COVID-19 severity and revealing the presence of neutrophils and macrophage cluster-1 as hallmarks of severe COVID-19. Further computational analyses identified several functional categories to be enriched in BAL from severe cases of infection, including inflammatory response, chemotaxis of myeloid cells, phagocytes, and granulocytes. Changes in BAL cells were further extrapolated to peripheral blood mononuclear cells (PBMCs) from a second cohort of COVID-19 patients. Deepening our understanding of the specific cell subsets responsible for severe outcomes in patients and expanding our knowledge on how these subsets contribute to the overall immune response according to their genes signatures will bring us closer to identifying possible therapeutic interventions for the treatment of COVID-19, in particular of those cases associated with severe complications.

## 2. Materials and Methods

### 2.1. Single-Cell Data Retrieval and Bioinformatics

A schematic presentation of the experimental and bioinformatics workflow for transcriptome analysis from BAL and PBMCs from COVID-19 patients and healthy subjects is provided in Supplementary Figure S1. Read count matrices (.H5) were retrieved from the SRP250732/GSM4475052 dataset. Expression values were first normalized to counts per ten thousand (CPTT) and were then subjected to the ICGS2 algorithm to identify cell types using Pearson correlation >0.2. Detailed description of the computational algorithm employed can be found in Venkatasubramanian et al. [15]. ICGS2 and AltAnalyze pipelines were found to outperform state-of-the-art scRNA-Seq detection workflows when applied to well-established benchmarks, as they combine multiple complementary subtype detection methods; Hierarchical Ordered Partitioning And Collapsing Hybrid (HOPACH, sparse non-negative matrix factorization, cluster ‘fitness’, support vector machine) to resolve rare and common cell states. ICGS2 identified cell clusters through a complex process of PageRank down-sampling, feature selection ICGS2, dimension reduction and clustering (sparse NMF, SNMF), cluster refinement (MarkerFinder algorithm), and finally cluster re-assignments using support vector machine (SVM). The top 500 genes with the highest dispersion were initially identified, followed by pairwise correlations of variable genes. Dimension reduction with sparse NMF was employed to improve the delineation of cell clusters following HOPACH clustering. The MarkerFinder algorithm was subsequently applied to identify rigorously defined cell clusters with unique gene expression for downstream cell cluster assignment, which identified genes that are positively correlated with an idealized cluster-specific expression profile. Cell cluster assignment was finally achieved from the marker genes identified for sufficiently fitting clusters, based on the cells assigned to the specific SNMF.

### 2.2. Gene Set Enrichment and Modeling of Gene Interactions Networks

Average gene expression levels across all cells (bulk) from each sample were subjected to comparative analysis (i.e., severe vs. healthy, severe vs. mild, and mild vs. healthy) using AltAnalyze v.2.1.3 pipeline. Differentially expressed genes were imported into the Ingenuity Pathways Analysis (IPA) software (Ingenuity Systems; www.ingenuity.com/) and were subjected to functional annotations and regulatory network analysis using upstream regulator analysis (URA) to analyze upstream molecules, which are connected to genes in the dataset via a set of either direct or indirect relationships with respect to their expression changes. Mechanistic networks (MN) takes URA further via the generation of plausible directional networks from these regulators, employing the IPA computational algorithm. Downstream effects analysis (DEA) identifies biological processes (disease) and functions, which are casually affected by the deregulation of genes in datasets and predicts the outline of biological process, whether upregulated or downregulated. IPA uses precise algorithms to predict functional regulatory networks from gene expression data and provides a significance score for each network according to the fit of the network to the set of focus genes in the database. The *p*-value is the negative log of P and represents the possibility that focus genes in the network are found together by chance [16,17].

### 2.3. RNA-Seq Analysis of PBMCs from COVID-19 Patients

Raw sequencing data (fastq) from COVID-19 PBMCs were retrieved from the SRP262058 dataset and were subsequently pseudo-aligned to the Gencode release 33 index; reads were subsequently counted using KALLISTO 0.42.1 [18], as we described before [19,20]. Normalized transcripts per million (TPM) expression values were subsequently subjected to differential analysis and hierarchical clustering as described before [21]. A volcano plot was used to illustrate the most differentially expressed genes (log 2-fold change) vs. log10 *p* value.

### 2.4. Statistical Analyses

Statistical analyses and graphing were performed using Microsoft excel 2016 and GraphPad Prism 8.0 software (GraphPad, San Diego, CA, USA). Two-tailed *t*-test was used for comparative groups; *p*-values ≤ 0.05 (two-tailed *t*-test) were considered significant. For IPA analyses, the activation z-score was utilized to infer the activation states of the indicated network and functional categories. A Z score of −2.0 ≥ Z ≥ 2.0 was considered significant.

## 3. Results

### 3.1. Single-Cell Transcriptome Analysis of BAL Revealed Variable Cellular Composition in Severe and Mild COVID-19 Patients Compared to Healthy Subjects

**Figure 1:**
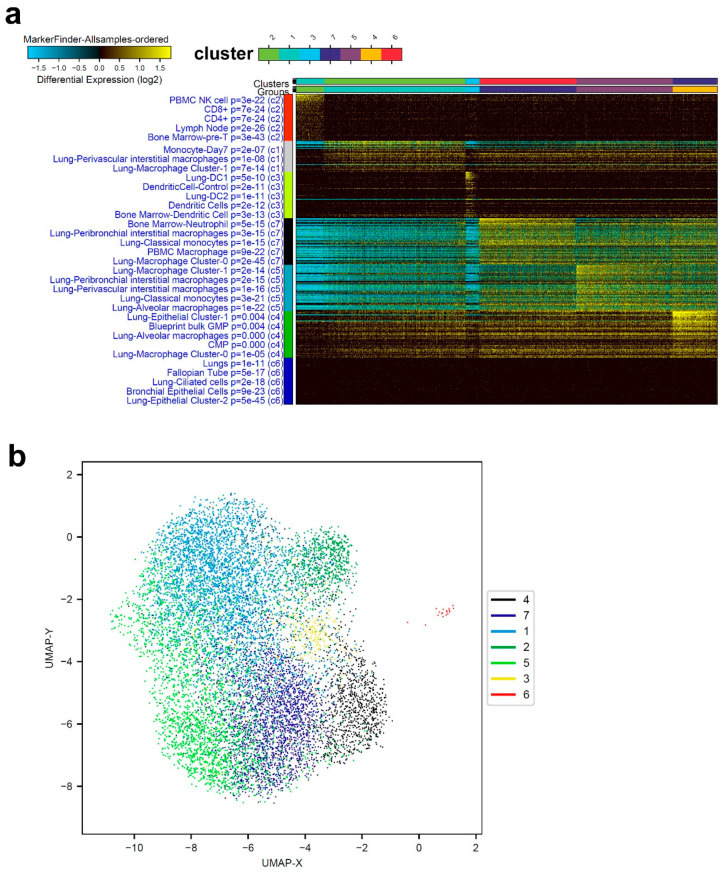
Representative single-cell analysis of bronchoalveolar lavage (BAL) from healthy subjects. (**a**) Representative single-cell analysis of BAL from healthy subjects employing the iterative clustering and guide-gene selection 2 (ICGS2) algorithm depicted as heat map. The text on the left indicates enriched cell-type markers from the default gene-set enrichment analysis and corresponding “Z” score *p* value. (**b**) Uniform manifold approximation and projection (UMAP) dimensionality reduction visualization of cell clusters corresponding to data presented in panel (**a**).

ICGS2 algorithm was employed to decipher the cellular composition of BAL from two healthy, three mild, and five severe COVID-19 patients. Clustering patterns from representative healthy subjects identified seven cell clusters enriched in T cells, lung macrophages (perivascular interstitial, Cluster-0, Cluster-1, alveolar), neutrophils, and dendritic cells (Figure 1a). Figure 1b displays an alternative visualization, dimensionally reducing the data from Figure 1a via UMAP. Cell clusters exhibited a significant overlap between different gene signatures of different cell populations from healthy subjects.

**Figure 2:**
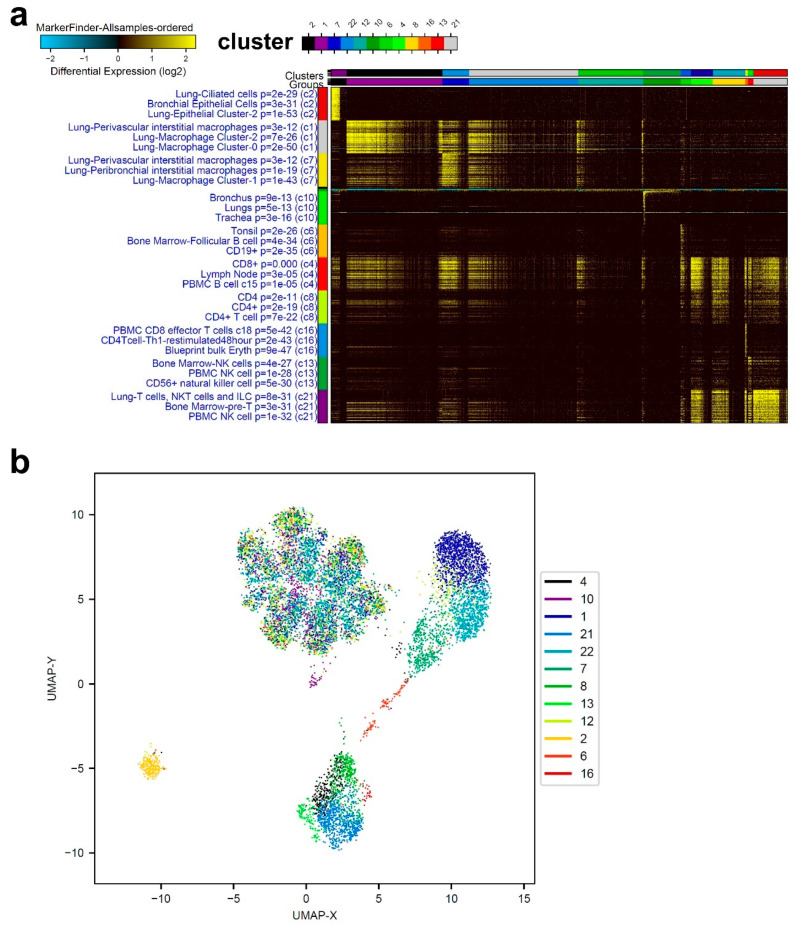
Representative single-cell analysis of BAL from a mild COVID-19 patient. (**a**) Representative single-cell analysis of BAL from a mild-COVID-19 patient employing the ICGS2 algorithm depicted as heat map. (**b**) UMAP dimensionality reduction visualization of cell clusters corresponding to data presented in panel (**a**).

ICGS2 analysis of patients with mild COVID-19 revealed distinct cell composition compared to the healthy control. Representative data from mild-COVID-19 BAL were mostly consistent of lung macrophages (cluster −0, −1, and −2), CD19, CD4, CD4 Th1, CD8, and NK cells, as well as lung and bronchial epithelial cells (Figure 2a). Figure 2b illustrates the data from a representative mild-COVID-19 patient using UMAP. Clusters 1, 2, 6, and 21 display significant distinction from other clusters, while the majority of other clusters were found centered around UMAP-X; 0, UPMA-Y; 5.

**Figure 3:**
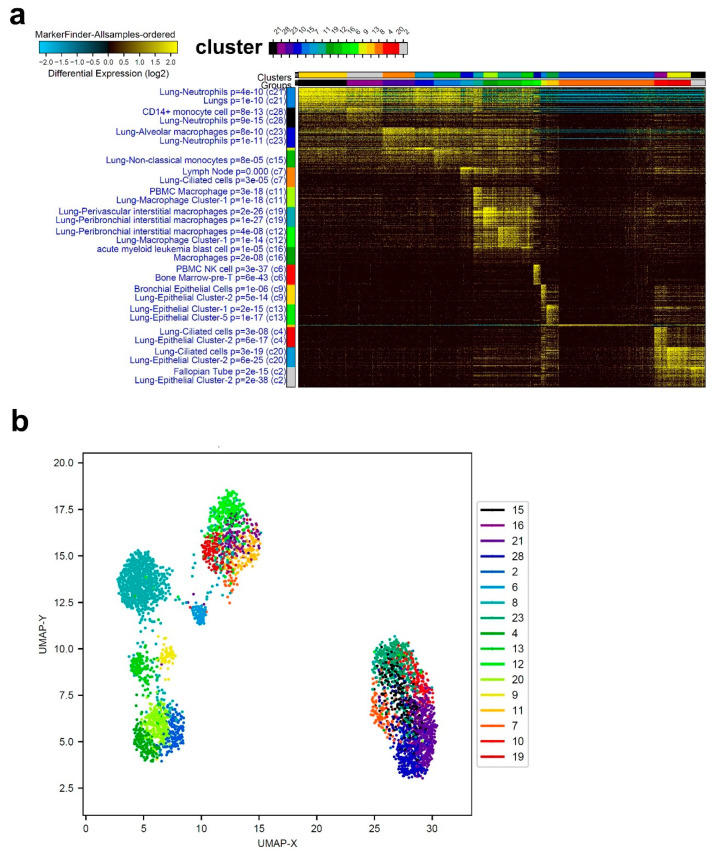
Representative single-cell analysis of BAL from a severe-COVID-19 patient. (**a**) Representative single-cell analysis of BAL from a severe COVID-19 patient employing the ICGS2 algorithm depicted as heat map. (**b**) UMAP dimensionality reduction visualization of cell clusters corresponding to data presented in panel (**a**).

We subsequently characterized the cellular composition from severe-COVID-19 patients. Single-cell analysis of a representative severe-COVID-19 patient highlighted a massive enrichment in neutrophils and macrophages, especially cluster-1 (Figure 3a). Similar to what found for mild-COVID-19 patients, BAL from severe-COVID-19 patients also included lung epithelial and bronchial cells. Top enriched markers in severe-COVID-19 BAL were spermidine/spermine N1-acetyltransferase 1 (SAT1), involved in the catabolic pathway of polyamine metabolism, LY8E, Spi-1 proto-oncogene (SPI1), Fc fragment of IgE, high-affinity I, receptor for gamma polypeptide involved in allergic reactions (FCER1G), and transforming growth factor beta 1 (TGFB1). A UMAP visualization of cell clusters corresponding to panel (a) is presented in Figure 1b.

### 3.2. Combination Analysis of Single-Cell Transcriptomes of BAL from Severe- and Mild-COVID-19 Patients Compared to Healthy Subjects

**Figure 4:**
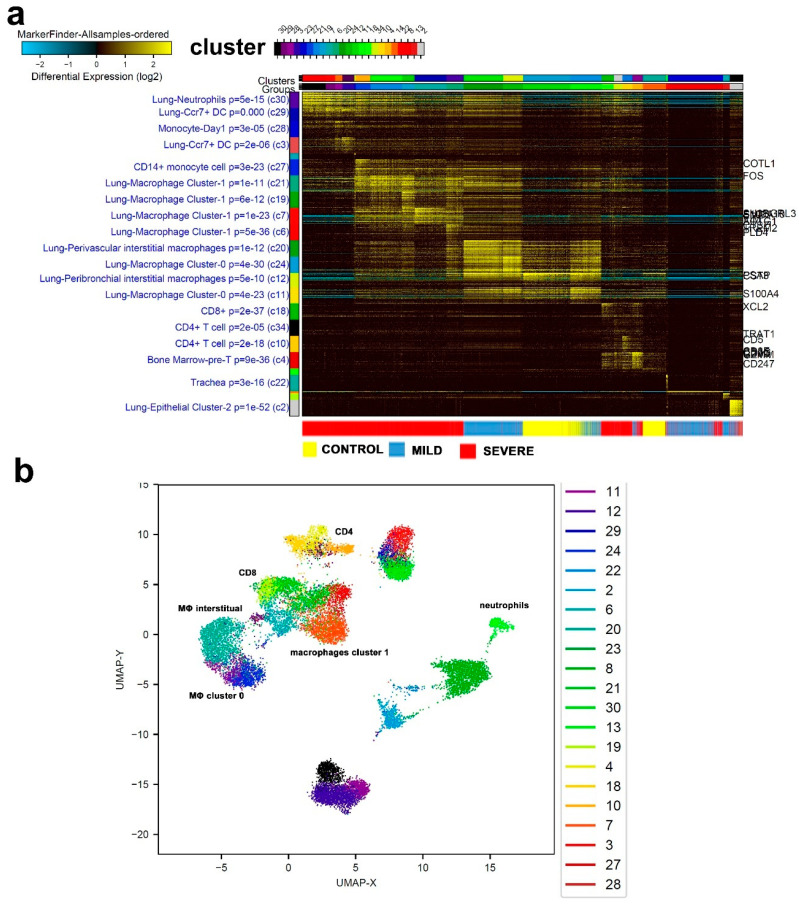
Combined single-cell analysis of BAL from severe- and mild-COVID-19 patients compared to healthy subjects. A total of 16,310 BAL-derived single cells from two healthy subjects, three patients with mild COVID-19, and five patients with severe COVID-19 were subjected to singl- cell analysis. Data are displayed as heat map (**a**) with the enriched cell population indicated on the left. (**b**) UMAP dimensionality reduction visualization of cell clusters corresponding to data presented in panel **a** with selected enriched cell populations indicated. MΦ: macrophages.

For a more comprehensive and comparative look into the single-cell transcriptome data and to avoid over-representation from different samples, equal number of single cells were randomly selected from each sample, hence, a total of 16,310 cells were subjected to ICGS2 analysis (Figure 4a, Supplementary Table S1). Firstly, we observed single cells from each of the three severity subsets to largely cluster together, with some overlap in the mild and severe cases. Whereas control and milder cases presented upregulation in gene markers associated with lung perivascular interstitial macrophages and lung macrophage cluster-0, single cells from the severe, and to a lesser extent, mild cases showed an upregulation in cell markers pertaining to lung neutrophils, lung CCR7, and dendritic cells (DCs), indicating a distinct immune response. Interestingly, severe-COVID-19 patients displayed a remarkable enrichment in neutrophils and macrophage cluter-1 compared to mild-COVID-19 patients and healthy controls. UMAP illustration of cell distribution from the same study group is shown in Figure 4b, with selected cell clusters being marked.

### 3.3. Enriched Gene Markers in Patients with Mild or Severe COVID-19 Compared to Healthy Subjects

**Figure 5:**
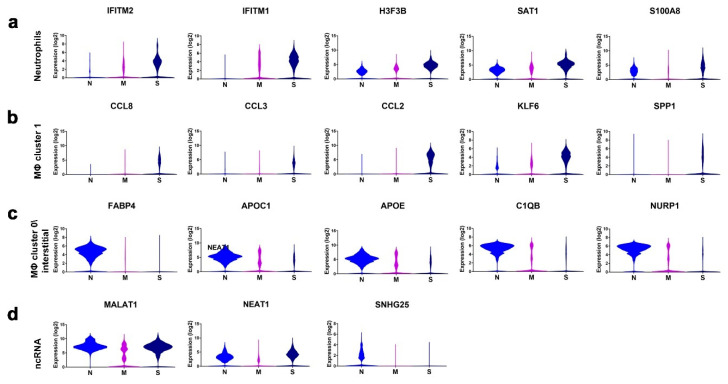
Expression of enriched gene markers in patients with mild or severe COVID-19 compared to healthy subjects. Expression of gene signatures derived from neutrophils (**a**), macrophage (MΦ) cluster 1 (**b**), macrophage cluster 0/interstitial (**c**), and noncoding RNAs (ncRNA) (**d**) in a total of 68,873 single cells derived from two healthy subjects, three patients with mild COVID-19, and five patients with severe COVID-19. Data are presented as violin plots. N: control, M: mild, S: Severe. Statistical analysis revealed significant differences in gene expression (*p* ≤ 0.0001) when comparing S vs. M, S vs. N, and M vs. N for the indicated genes.

The ICGS2 algorithm identified gene signatures with the strongest correlation with the indicated cell types. We subsequently explored the expression of selected gene markers indicative of neutrophils, macrophage (MΦ) cluster 1, macrophage cluster 0/interstitial, and noncoding RNAs (ncRNAs) in a total of 68,873 single cells derived from the same cohort of patients with varying COVID-19 severity. Representative gene signatures of each cell population are shown in Figure 5a–d. Neutrophil-derived signature (*IFITM2*, *IFITM1*, *H3F3B*, *SAT1*, and *S100A8*) exhibited significantly higher expression (*p* < 0.0001) in severe cases compared to normal, with the mild cases exhibiting intermediate expression between the normal and the severe cases (Figure 5a). The macrophage cluster-1-derived signature (*CCL8*, *CCL3*, *CCL2*, *KLF6*, and *SPP1*) was confirmed to be significantly higher in severe cases of COVID-19 compared to control and mild cases (Figure 5b). Patients with devere and mild COVID-19 exhibited substantial reduction in the expression of *FABP4*, *APOC1*, *APOE*, *C1QB*, and *NURP1*, all associated with interstitial and macrophage cluster-0 (Figure 5c). *MALAT1*, *NEAT1*, and *SNHG25* long noncoding RNAs (lncRNAs) were downregulated in mild and severe COVID-19 BAL cells. Further downregulation of *MALAT1* and *NEAT1* was observed in mild-COVID-19 BAL cells, while *SNHG25* was markedly downregulated in both mild- and severe-COVID-19 BAL cells (Figure 5d).

### 3.4. Canonical and Upstream Regulator Pathway Analyses Highlighted Activation of Innate Immune Response in BAL Cells from Severe COVID-19

**Figure 6:**
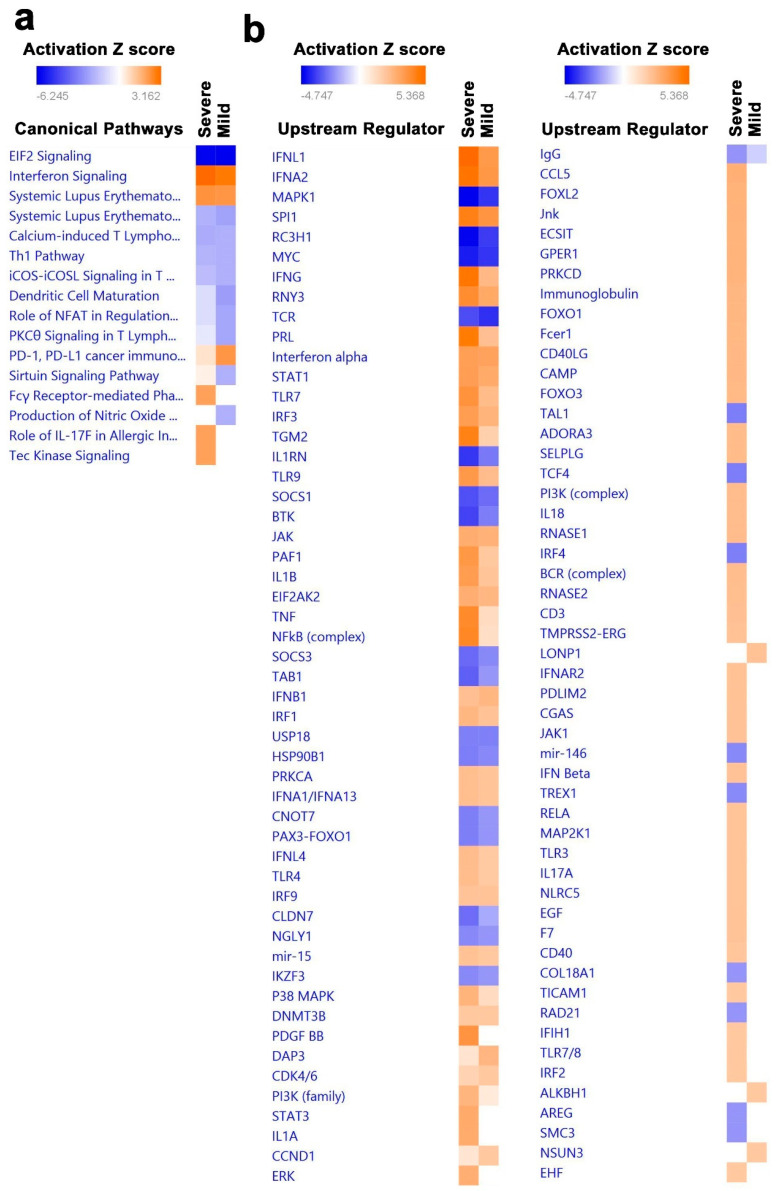
Identification of canonical pathways and upstream regulator networks associated with severe and mild COVID-19. Differentially expressed genes in patients with severe vs. control and mild vs. control COVID-19 were subjected to canonical and upstream regulator analysis using ingenuity pathways analysis (IPA). (**a**) Comparative analysis of significantly altered canonical pathways in mild- and severe-COVID-19 BAL transcriptome data. (**b**) Comparative analysis of significantly altered upstream regulatory networks in mild- and severe-COVID-19 BAL transcriptome data. Red indicates activated, while blue indicates suppressed pathways. Activation Z score is depicted according to the color scale.

The average gene expression levels across all cells in each sample were subjected to differential expression analysis comparing patients with severe (Supplementary Table S2) and mild COVID-19 (Supplementary Table S3) to healthy subjects. Differentially expressed genes were subsequently subjected to comparative canonical pathway analysis in IPA, revealing modest enrichment in a number of canonical pathways including innate immunity associated with interferon signaling, FC gamma, IL17, and Tec kinase signaling in severe-COVID-19 BAL cells, while PD-1 and PDL-1 pathways were suppressed (Figure 6a). Furthermore, pathways associated with adaptive immune responses were predominantly signaling pathways that contribute to the regulation of activated effector T-cell functions such as iCOS–iCOSL, Th1, protein kinase C-theta (PKC theta), calcium-induced T lymphocyte apoptosis and dendritic cell maturation pathways, which were downregulated in both severe- and mild-COVID-19 BAL cells (Figure 6a). Similarly, an in-depth comparative analysis of severe- and mild-COVID-19 BAL transcriptome data showed significant enrichment of several upstream regulators. In particular, IFNG, PRL, TGM2, TLR9, PAF1, IL1B, TNF, and NFKB were activated in severe-COVID-19 BAL cells (Figure 6b). However, several other upstream regulators were suppressed in severe- and mild- COVID-19 BAL cells compared to healthy individuals.

**Figure 7:**
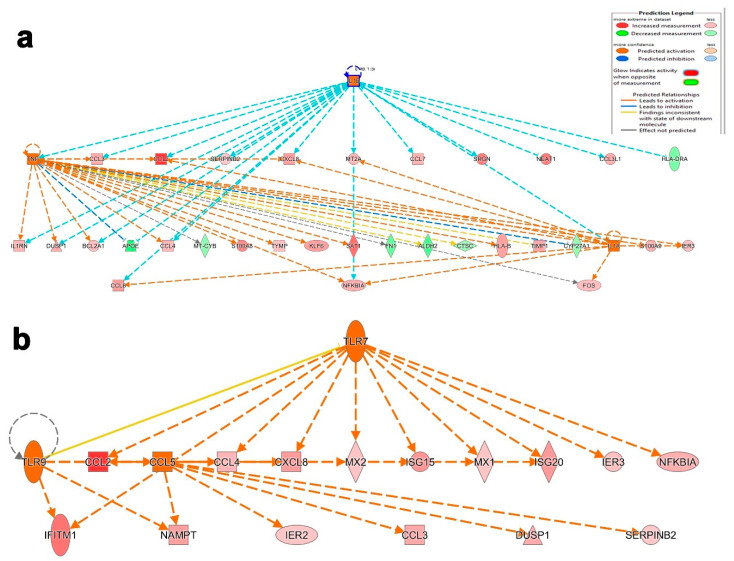
Illustration of selected activated upstream networks in patients with severe COVID-19. Graphical presentation of IL1A, IL1B, and TNF (**a**) networks in patients with severe COVID-19. (**b**) Presentation of CCL5, TLR7, and TLR9 upstream regulator networks.

We subsequently sought to identify the pathways and functional categories unique to severe-COVID-19 BAL cells. Differential gene expression analysis of BAL cells from severe- compared to mild-COVID-19 patients revealed 53 upregulated and 34 downregulated genes (Supplementary Table S4). Mechanistic network analysis elucidates the pragmatic alterations in appropriate gene expression through upstream regulator prediction. The activated mechanistic networks in patients with severe COVID-19 highlighted the predicted relationship between IL1A, IL1B, and TNF, and their regulation in gene datasets based on Z scores. Herein, the main IL1B upstream regulator activated 16 downstream molecules including two upstream regulators, TNF and IL1A, and inhibited two downstream genes, *HLA-DRA* and *APOE*. Similarly, TNF activated 16 and inhibited 6 downstream target genes, among those 9 genes (upregulated *CCL3, CCL4, ILR1, DUSP1, BCL2A1* and downregulated *APOE*) including IL1A upstream regulator, which were common between IL1B and TNF. Furthermore, the upstream regulator IL1A was predicted to activate seven downstream targets, among which, four shared with IL1B (M2A, CXCL8, CXCL2, TNF, and CCL8) and two shared with TNF (NFKBIA and FOS) (Figure 7a). Similarly, another mechanistic network illustrates the relationship between CCL5, TLR7, and TLR9. Mainly, the upstream regulator TLR7 was predicted to activate 11 downstream targets including two upstream regulators, TLR9 and CCL5. Further analysis with TLR9 revealed direct activation of two downstream targets (IFITM1 and NAMPT) shared with CCL5 and another target, ISG20, which is shared with TLR7 through seven intermediated targets, including the main upstream regulator CCL5. Interestingly, CCL5 is predicted to exert the same stimulatory effect on CCL2 as TLR9 and TLR7 and have distinct effects on four more downstream targets (Figure 7b).

### 3.5. Disease and Functional Analysis of Differentially Expressed Genes in BAL from Severe- and Mild- COVID-19 Patients Indicated the Activation of Innate Immune Responses

**Figure 8:**
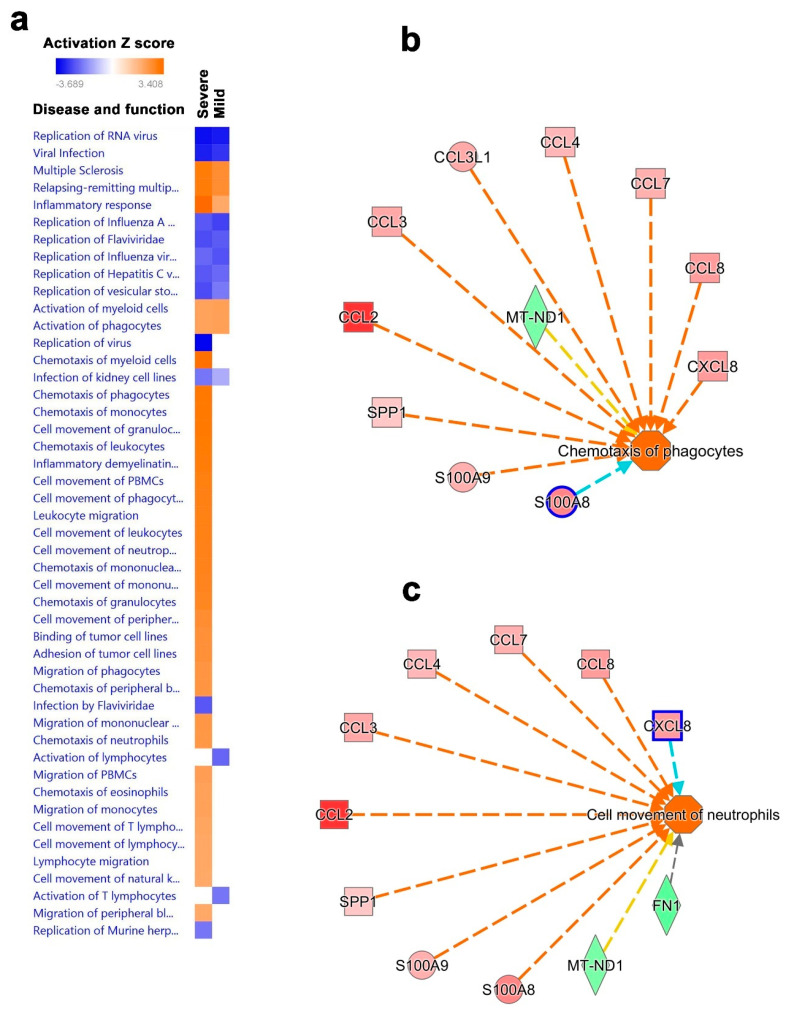
Disease and functional analysis of differentially expressed genes in BAL from patients with mild and severe COVID-19. (**a**) Heat map depicting the activation states of the indicated disease and function categories in mild- and severe-COVID-19 BAL cells. Illustration of chemotaxis of phagocytes (**b**) and cell movement of neutrophils (**c**) in patients with severe COVID-19 based on IPA analysis.

In order to understand the downstream functional effects of upstream regulators in severe- and mild-COVID-19 BAL cells, we employed IPA downstream effector analysis. Our analysis predicted the various disease and functional activities based on the deregulated upstream regulator molecules in the dataset portrayed as a vertical heat map. Comparative analysis revealed common deregulated disease activities and functions in BAL cells from patients with severe and mild COVID-19, including the downregulation of RNA virus replication, viral infection, replication of influenza, and hepatitis C and vesicular stomatitis virus, and the upregulation of multiple sclerosis inflammatory response and activation of myeloid cells and phagocytes (Supplementary Table S5). Comparatively, many disease and functional pathways were upregulated only in severe-COVID-19 BAL cells, including chemotaxis, cell adhesion, cell movement, migration of myeloid cells, phagocytes, granulocytes, monocytes, and cell movement of NK cells. Interestingly, activation of total lymphocyte and T lymphocyte pathways were downregulated. Therefore, our downstream effector analysis revealed the augmentation of innate immune responses in severe-COVID-19 patients (Figure 8a). Disease and function analysis of BAL from severe- vs. mild-COVID-19 patients revealed a number of activated functional categories (Supplementary Table S6). Of particular interest, our regulator effector analysis in severe-COVID-19 BAL cells revealed activation of CCL2, CCL3, CCL3L1, CCL4, CCL7, CCL8, CXCL8, SPP1, S100A8, and S100A9, and inhibition of MT-ND1 and FN1 upstream regulators which is predicted to trigger the chemotaxis of phagocytes and cellular movement of neutrophils in severe-COVID-19 BAL cells (Figure 8b,c).

### 3.6. Similarities in Transcriptome Data from BAL and PBMCs from COVID-19 Patients

**Figure 9:**
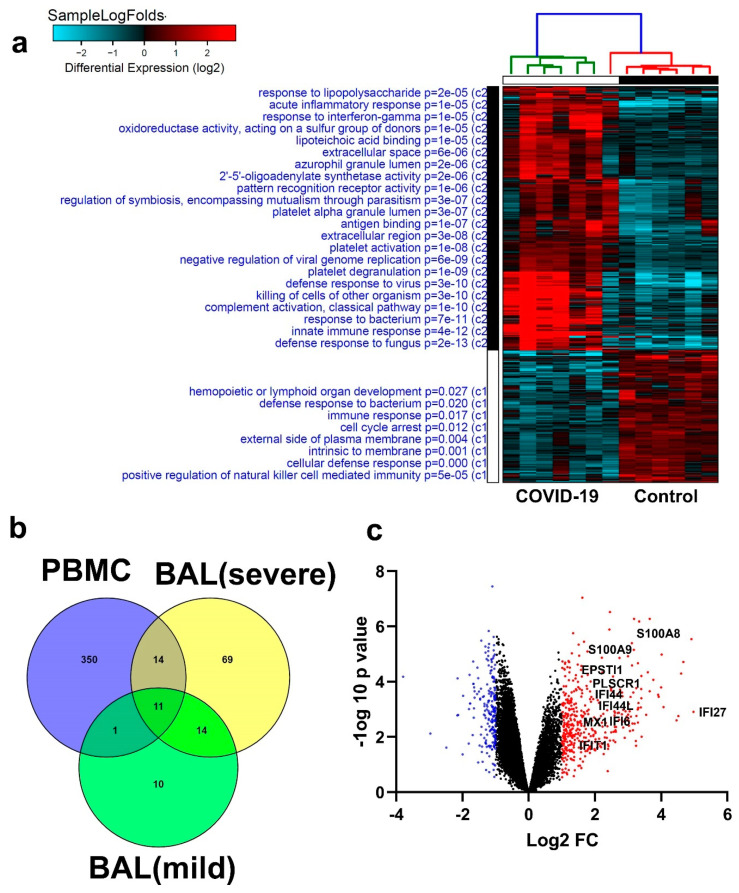
Similarities in transcriptome data from BAL and peripheral blood mononuclear cell (PBMCs) in COVID-19 patients. (**a**) Hierarchical clustering summarizing differential gene expression (log2) in PBMCs from COVID-19 and normal controls, highlighting enriched functional categories (GO). (**b**) Venny diagram illustrating the aberrantly expressed genes in common in severe- and mild-Covid-19 BAL and PBMC, with 11 genes in common to the three categories. (**c**) Volcano plot depicting selected genes common to BAL and PBMCs, indicating upregulated (red) and downregulated (blue) genes.

To further highlight if the observed changes in BAL cells can be reflected in the circulation, we explored PBMCs transcriptome data from an independent cohort of seven COVID-19 patients and six healthy controls, revealing commonalities for several genes differentially expressed in BAL and PBMCs. Hierarchical clustering based on differential gene expression (log2) (Figure 9a and Supplementary Table S7) highlighted the enrichment in functional categories (GO) involved in defense responses to viruses, killing cells of other organisms, and activation of innate (complement) immune functions and classical pathways in PBMCs from COVID-19 patients. Other upregulated functions in COVID-19 patients included processes related to acute inflammatory responses, response to interferon gamma, pattern recognition receptor activity, antigen binding, and platelet activation, as well as negative regulation of viral genome replication. On the other hand, the most enriched functional category in the healthy group was positive regulation of NK cell-mediated immunity, suggesting a possible suppression of this cell population in the periphery of COVID-19 patients. Interestingly, when comparing PBMCs and the BAL-derived data from the two independent cohorts, we observed 11 genes (*IFI27*, *IFITM3*, *IFI6, ISG15*, *IFIT3*, *RSAD2*, *MX1*, *IFIT1*, *OASL*, *IFI30*, and *OAS1*) that were found in all three categories, PBMCs, BAL from severe-COVID-19 patients, and BAL from mild-COVID-19 patients. In addition, there were 14 commonly upregulated genes (*S100A8*, *IFI44L*, *IFI44*, *CXCL8*, *CCR1*, *PLSCR1*, *EPSTI1*, *FPR1*, *OAS2*, *IL1RN*, *TYMP*, *BCL2A1*, *GAPDH*, and *OAS3*) in severe-Covid-19 BAL cells and PBMCs, and one gene in common (*C1QC*) for BAL and PBMCs form mild cases of COVID-19. (Figure 9b). Volcano plots depicting selected genes common to BAL and PBMCs, indicating the upregulated (red) and downregulated (blue) genes, are shown in Figure 9c. The upregulated genes included *S100A8, S100A9, IFI27, EPSTI1*, and *EPSTI1*. These genes encode calcium-binding proteins that play important roles in the regulation of inflammatory immune responses. Constitutively expressed in neutrophils and monocytes, they could lead to the induction of neutrophil chemotaxis and adhesion. Such commonalities displayed in BAL and in the circulation could provide us with potential biomarkers to target in the development of therapeutic interventions against viral infections.

## 4. Discussion

BAL has been widely used in diagnosing lower respiratory airway infections [22] and has recently provided us with data on the pulmonary microenvironment during COVID-19 infection. Utilizing single-cell gene expression data from patients with COVID-19 at varying severity in combination with modern computational analysis identified rigorously defined cell clusters, revealing the presence of neutrophils and macrophages cluster-1 as hallmarks of severe COVID-19. Our data are in agreement with the work of Liao et al., who reported the presence of proinflammatory monocyte-derived macrophages in the BAL fluid from patients with severe COVID-9 employing the same dataset [2]. Gene signatures highlighted interferon-induced transmembrane (IFITM) protein 1 and 2 (*IFITM1* and *IFITM2*), which have been associated with other viruses including influenza and West Nile Virus [23,24]. We recently reported on the upregulation of several IFITM family members in bronchial epithelial cells infected with SARS-CoV-2 [25]. The involvement of such gene signatures emphasizes the link between innate immune response and effects of COVID-19 on interferon gamma signaling. Histone structure and regulation also play a key role in the gene regulation of any transcript. Modifications in histones such as HAT1, HDAC2, and KDM5B were revealed by network analysis, identifying these histones as potential regulators of the SARS-CoV-2 receptor ACE2 in the human lung. In a cohort of 700 lung transcriptome samples, increased expression of ACE2 and the effect of histone protein modifications in these patients are suggested to induce a severe COVID-19 phenotype [26]. H3 histone, family 3B (H3F3B), associated with neutrophils in our data, has been described in several cancers such as chondroblastoma [27] and hepatocellular carcinoma [28] and in ovarian cancer cell lines [29], where point mutations or upregulation in expression of its corresponding gene caused dysregulations and transcriptional changes leading to disease onset and progression. Another study showed that CCL8 was detected at high levels in the peritoneal fluid of patients who exhibited anastomotic leakage after colorectal surgery [30]. Elevated levels of *SPP1* expression were also found to correlate with highly aggressive lung adenocarcinoma [31]. Zuo et al. described the role of neutrophil extracellular traps (NETs) released by neutrophils in order to regulate infection; however, when in excess, they contribute to inflammation and cytokine release, leading to thrombosis in the lungs and respiratory failure in patients with severe COVID-19 [32]. Such evidence backs our findings and confirms the associations between these gene signatures and processes such as the involvement of neutrophils and macrophages in inflammation in several disease types, including viral infection.

Pathological changes are associated with increased vascular permeability induced by the binding of SARS-CoV-2 to ACE2 receptors on endothelial cells, followed by the recruitment of activated neutrophils, macrophages, and other immune cells, which collectively result in increased production of inflammatory cytokines including IL-6, IL-8, G-CSF, MCP1, IL-2, TNF-α, and IL-1β [33,34]. Some of these cytokines further amplify the inflammatory loop and induce the recruitment of more inflammatory cells, while others initiate and activate the coagulation-mediated cascade [33]. In turn, persistent unresolved inflammation leads to endothelial cell dysfunction, Disseminated intravascular coagulation (DIC), alveolar dysfunction, severe acute respiratory distress syndrome (ARDS), and ultimately multi-organ failure and death [33,35]. Higher levels of several inflammatory cytokines including TNF-α, IL-6, and IL-1 and inflammatory chemokines such as CCL2, CCL3, and CXCL10 are associated with disease severity and death in COVID-19 patients [36]. TNF-α and IL-1β induce vasodilation and permeability, which allows immune cells to reach the sites of damage, while IL-β and IL-6 induce complement and opsonization. Different chemokines and cytokines, including CCL2, 3, 5, CXCL8, 9, 10, IFNγ, TNFα, IL-1-β, IL-1RA, IL-6, IL-7, IL-8, IL-12, IL33, GCSF, GMCSF, IP10, MCP1, MIP1α, MIP1β, PDGFB, and VEGFA, contribute to cytokine storms [37,38]. These chemokines are primarily involved in the recruitment of other leukocytes to tissues, while pro-inflammatory cytokines are involved in effector functions causing damage to cells [39,40]. The resulting intense immune response is extensively documented in ARDS affecting the lungs but leads to multi-organ dysfunction (MODS) and failure via tissue damage and ultimately to death in severe SARS-CoV-2 infections. In line with this, high cytokine levels have been reported in critically ill COVID-19 patients [9].

Ingenuity pathway analysis revealed enrichment of interferon signaling, suggested by Felgenhauer et al. to be a potential key player in the management of COVID-19, since IFNs type I and III inhibited SARS-CoV-2 in a dose-dependent manner [41]. In other reports, increased IFN type I and II production in response to viral infection was found to impair lung epithelial regeneration throughout the duration of recovery from viral infection [42]. The idea of targeting Fcγ receptor (FcγR) pathways identified in our ingenuity pathway analysis is in agreement with a study by Chakraborty et al., which reported a global analysis of antibodies produced during SARS-CoV-2 infections [43]. Reduced Fc glycosylation in COVID-19 patients led up to a 10-fold higher affinity for FcγRIIIa, which is abundant on monocytes, macrophages, and NK cells, in turn promoting pro-inflammatory cytokine production and cytotoxic effector cell activity [44]. Activation of such pathways could be a contributing factor to severe COVID-19 and provide potential biomarkers for targeted therapy.

Other functional categories in BAL associated with severe-COVID-19 patients include inflammatory response and chemotaxis of myeloid, phagocytes, and granulocytes, among the most activated. This is expected in these cases, as viral recognition by macrophages initiates the recruitment of other immune cells through IL-6, TNF-α, IL-1β, and type-1 interferon signaling [36].

Emerging evidence highlights the roles played by lncRNAs during the course of viral infection. Gene signatures from three lncRNA gene markers (*MALAT1*, *NEAT1*, and *SNHG25*) were found to be downregulated in mild and severe cases of COVID-19, compared to normal controls. Studies on MALAT1 in inflammatory injury following lung transplant interestingly showed that the silencing of *MALAT1* alleviated inflammatory injury by inhibiting neutrophil chemotaxis and immune cell infiltration to the site of infection [45]. The downregulation of *MALAT1* in our analysis could indicate a role for this lncRNA as an agent for the regulation of neutrophil chemotaxis that is rife in severe cases, in efforts to naturally alleviate inflammatory injury in COVID-19-positive cases. NEAT1 lncRNA has also been associated with viral infection, namely, HIV-1. Its knockdown, as shown by Zhang et al., led to enhanced viral production and inflammation by promoting the export of HIV-1 mRNA transcripts in HeLa cells [46].

Interestingly, when comparing data from BAL to those from PBMCs in the circulation, several genes were identified in common as aberrantly expressed, highlighting the potential of using PMBCs in the circulation as liquid biopsies in order to identify initial clues surrounding the immune microenvironment upon infection. Several of the commonly upregulated genes in BAL and PBMCs from COVID-19 patients were indicative of an interferon response. Upregulated genes included *S100A8*, *S100A9*, *IFI27*, *EPSTI1*, and *EPSTI1*. These genes encode calcium-binding proteins that play an important role in the regulation of inflammatory immune responses. Constitutively expressed in neutrophils and monocytes, they could lead to the induction of neutrophil chemotaxis and adhesion, as we have previously reported in Calu-3 human lung epithelial cells [47]. Our data are concordant with those of Wilk and colleagues who also reported the presence of an interferon-stimulated gene signature and neutrophils in the circulation of patients with acute respiratory failure requiring mechanical ventilation [14]. Such commonalities displayed by BAL and the circulation could provide us with potential biomarkers to target in the development of therapeutic interventions against viral infections.

Taken together, our data revealed the presence of neutrophils and macrophage cluster-1 as the main immune cell subsets associated with severe COVID-19 and identified their inflammatory and chemotactic gene signatures, as well as possible upstream regulators and potentially affected mechanistic networks throughout the course of SARS-CoV-2 infection. We also identified commonalties in transcriptome data from BAL and PBMCs in COVID-19 patients. Further functional studies are needed to expand our understanding of how neutrophils and macrophage cluster-1 specifically affect the immune system and of the downstream consequences this has upon SARS-CoV-2 infection. However, this study provides an interesting introduction for the potential identification of possible immune-based therapeutic interventions.

## Supplementary Materials

The following are available online at https://www.mdpi.com/2073-4409/9/11/2374/s1, Figure S1: Schematic presentation of the experimental and bioinformatics workflow for transcriptome analysis of BAL and PBMCs from COVID-19 patients and healthy subjects, Table S1: Marker genes associated with the indicated cell clusters in healthy, mild- and severe-COVID-19 BAL cells, Table S2: Differentially expressed genes in BAL cells from mild-COVID-19 patients vs. healthy subjects, Table S3: Differentially expressed genes in BAL cells from severe-COVID-19 patients vs. healthy subjects, Table S4: Comparison of upstream analysis of BAL cells from severe- COVID-19 patients vs. control and mild- COVID-19 patients vs. control, Table S5: Disease and function comparison between severe and mild cases of COVID-19, Table S6: Comparison of disease and function between severe-Covid-19 patients vs. control and mild- Covid-19 patients vs. control, Table S7: Differentially expressed genes in PBMCs from COVID-19 patients vs. control.
