# Supplementary material for: Single-Cell Transcriptome Analysis Highlights a Role for Neutrophils and Inflammatory Macrophages in the Pathogenesis of Severe COVID-19
Source: Cells. 2020 Oct 29;9(11):2374. doi: 10.3390/cells9112374 (PMC7693119; doi:10.3390/cells9112374)
Supplement: Supplementary file 1 [file cells-09-02374-s001.zip › Supplementary figure 1.pdf]

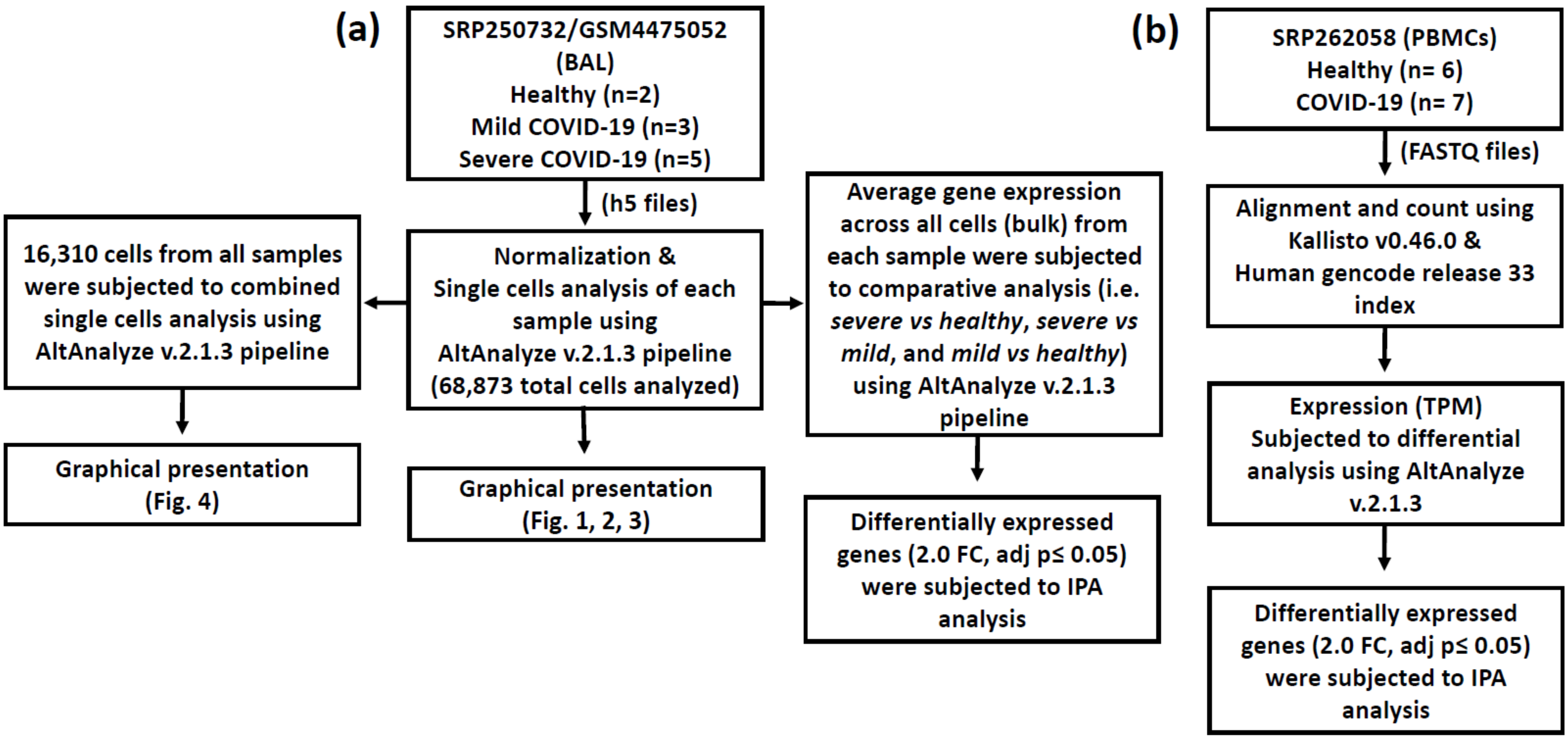

Supplementary figure 1. Schematic presentation of the experimental and bioinformatics workflow for transcriptome analysis from Bronchoalveolar lavage (BAL, a) and peripheral blood mononuclear cells (PBMCs) from COVID-19 patients and healthy subjects.
